# Continuing Reassortant of H5N6 Subtype Highly Pathogenic Avian Influenza Virus in Guangdong

**DOI:** 10.3389/fmicb.2016.00520

**Published:** 2016-04-13

**Authors:** Runyu Yuan, Zheng Wang, Yinfeng Kang, Jie Wu, Lirong Zou, Lijun Liang, Yingchao Song, Xin Zhang, Hanzhong Ni, Jinyan Lin, Changwen Ke

**Affiliations:** ^1^Key Laboratory for Repository and Application of Pathogenic Microbiology, Research Center for Pathogens Detection Technology of Emerging Infectious Diseases, Guangdong Provincial Center for Disease Control and PreventionGuangzhou, China; ^2^WHO Collaborating Centre for Surveillance, Research and Training of Emerging Infectious DiseaseGuangzhou, China; ^3^Key Laboratory of Zoonosis Prevention and Control of Guangdong, College of Veterinary Medicine, South China Agricultural UniversityGuangzhou, China; ^4^School of Public Health, Sun Yat-Sen UniversityGuangzhou, China

**Keywords:** reassortant, highly pathogenicity, avian influenza virus, H5N6, live poultry market

## Abstract

First identified in May 2014 in China's Sichuan Province, initial cases of H5N6 avian influenza virus (AIV) infection in humans raised great concerns about the virus's prevalence, origin, and development. To evaluate both AIV contamination in live poultry markets (LPMs) and the risk of AIV infection in humans, we have conducted surveillance of LPMs in Guangdong Province since 2013 as part of environmental sampling programs. With environmental samples associated with these LPMs, we performed genetic and phylogenetic analyses of 10 H5N6 AIVs isolated from different cities of Guangdong Province from different years. Results revealed that the H5N6 viruses were reassortants with hemagglutinin (HA) genes derived from clade 2.3.4.4 of H5-subtype AIV, yet neuraminidase (NA) genes derived from H6N6 AIV. Unlike the other seven H5N6 viruses isolated in first 7 months of 2014, all of which shared remarkable sequence similarity with the H5N1 AIV in all internal genes, the PB2 genes of GZ693, GZ670, and ZS558 more closely related to H6N6 AIV and the PB1 gene of GZ693 to the H3-subtype AIV. Phylogenetic analyses revealed that the environmental H5N6 AIV related closely to human H5N6 AIVs isolated in Guangdong. These results thus suggest that continued reassortment has enabled the emergence of a novel H5N6 virus in Guangdong, as well as highlight the potential risk of highly pathogenic H5N6 AIVs in the province.

## Introduction

Depending on their pathotypes, avian influenza viruses (AIVs) have inherently different pathogeneses in the infection and distribution of lesions. According to pathotype, AIVs can divide into non-pathogenic AIVs (NPAIV), low pathogenic AIVs (LPAIV), and highly pathogenic AIVs (HPAIV). In birds, NPAIVs (e.g., H6N6) usually present no clinical symptoms; by contrast, LPAIVs (e.g., H9N2) can cause mild respiratory or gastrointestinal infection, and HPAIVs (e.g., H5N1 and H7N2) can induce systemic, multi-organ infection, as well as high morbidity, and mortality (Swayne and Halvorson, 2003; Yuan et al., 2014).

A continual threat to animal and human health, HPAIVs have caused infections and deaths in not only countless birds, but also many humans. In 1997, the H5N1 HPAIV, the internal genes of which derived from the H6N1 NPAIV, infected 18 people in Hong Kong, six of whom died from the infection (Claas et al., 1998; Subbarao et al., 1998). Furthermore, since 2003, the H5N1 HPAIV has caused outbreaks both in birds and humans in more than 60 countries, including China (Yuan et al., 2014; WHO, 2015a). Recently, H5-subtype HPAIV s—that is, variants of different NA subtypes—have also caused outbreaks in poultry in China (i.e., subtypes H5N1, H5N2, H5N5, H5N6, and H5N8), as well as in South Korea (i.e., subtype H5N8), Japan (i.e., subtype H5N8), Laos (i.e., subtype H5N8), and Vietnam (i.e., subtypes H5N1 and H5N6; WHO, 2014d; OIE, 2015). In March 2014, an outbreak of H5N6 HPAIV in poultry was reported in Laos and, that April, in Vietnam (Wong et al., 2015). Genetic studies have shown that the H5N6 virus has exchanged genes from the H5N1 and H6N6 AIVs that circulate widely in ducks (Shen et al., 2015). Although little is known about the potential of these novel viruses to infect humans, a few isolated cases have been detected. On May 6, 2014, one such case of H5N6 infection in China's Sichuan Province was fatal (CDC China, 2014; WHO, 2014c), and later that year, another severe case of infection occurred in Guangdong Province in December (WHO, 2014a). As of February 2016, nine cases of H5N6 AIVs infection in humans have been confirmed in China, six of them in Guangdong Province (WHO, 2015b,c, 2016a,b,c).

Since 2013, several surveillance systems for pandemic preparedness have been established in China, including those at live poultry markets (LPM) and sentinel hospitals. These surveillance systems have played a vital role in the early detection of warning signs of AIV infection in humans. During our study's surveillance period, we isolated 10 H5N6 AIVs in environmental samples from LPMs in Guangdong Province, and to better understand their genetic diversity and evolution, we analyzed their related epidemiological and sequence data.

## Materials and methods

### Ethics statement

This research was reviewed and approved by the South China Agricultural University Experimental Animal Welfare Ethics Committee (permit no. 2014-11).

### Sample collection

Beginning on April 16, 2013, in order to better monitor LPMs for AIV contamination and assess the risk of AIV infection in humans, environmental sampling programs were implemented in Guangdong Province. Environmental samples were taken from poultry excrement, epilator swabs, and sewage swabs—the latter two from drains in meat preparation areas or around cages—whereas chopping swab samples were gathered randomly from butcher boards or knives at LPMs each week.

### Virus isolation

Samples were first tested for influenza A by using real-time polymerase chain reactions (qPCR) in the laboratories of the district's Centers for Disease Control and Prevention (CDC). Positive influenza A samples were probed to detect subtypes H5, H7, and H9 by using qPCR in local CDC laboratories, and results were later verified by Guangdong's CDC. H5-positive samples were further analyzed by using qPCR to detect the presence of the N6 gene. All qPCR-detected primers and probes were provided by the Chinese CDC. Samples positive with H5N6 subtypes were purified and propagated in 10-d embryonated chicken eggs free of specific pathogens and stored at −70°C until used. Subtypes of the viruses were further identified by hemagglutination (HA) inhibition assay. All experiments were carried out in animal biosafety level 3 facilities.

### Genomic sequencing

Viral RNA was first extracted from allantoic fluid by using an RNA extraction kit (QIAamp Viral RNA Mini Kit, Qiagen, Hilden, Germany). Reverse transcription and polymerase chain reaction (PCR) amplification of all eight gene segments used pre-amplification reagents (PathAmp™ FluA, Life Technologies, Guilford, Connecticut, USA). PCR products were purified and quantified with a purification kit (AmpureXP, Beckman Coulter, Porterville, CA, USA) according to the manufacturer's instructions. The full genomes of the viruses were sequenced with a sequencing kit (Ion PGM Sequencing 200 Kit version 2, Life Technologies), specifically with the kit's Ion 316 Chip V2 and according to the manufacturer's instructions.

### Sequence analysis

To align and analyze the sequences, multiple sequences of the representative AIVs were downloaded from GenBank databases (Li et al., 2010; Yuan et al., 2014, 2016). Full-length gene sequences were implemented and edited with Lasergene 7.1 (DNASTAR, Madison, Wisconsin, USA). A neighbor-joining algorithm and maximum-likelihood trees model were estimated for all eight genes—namely, HA, NA, PB2, PB1, PA, NP, M, and NS—by using genetic analysis software [Molecular Evolutionary Genetics Analysis (MEGA) version 6.06] with 1000 bootstrap trials. Branches with bootstrap values exceeding 50% were grouped together in the trees.

Nucleotide sequences obtained in our study, all listed by their accession numbers, are currently available from GenBank (Table 1).

**Table 1:** **Isolation of H5N6-subtype avian influenza viruses from live poultry markets in Guangdong, 2013–2015**.

**Virus**	**Abbreviation**	**Collection city**	**Collection date**	**Accession number (PB2, PB1, PA, HA, NP, NA, M, NS)**
A/Environment/Guangdong/QY025/2013(H5N6)	QY025	Qingyuan	2013.04	KT370109, KT370102, KT370095, KT370059, KT370082, KT370076, KT370067, KT370088
A/Environment/Guangdong/JY137/2014(H5N6)	JY137	Jieyang	2014.03	KT370111, KT370105, KT370098, KT370064, KT370084, KT370074, KT370071, KT370092
A/Environment/Guangdong/PY955/2014(H5N6)	PY955	Guangzhou	2014.12	KT370113, KT370106, KT370099, KT370062, KT370079, KT370075, KT370069, KT370091
A/Environment/Guangdong/QY197/2014(H5N6)	QY197	Qingyuan	2014.05	KT370108, KT370101, KT370094, KT370060, KT370081, KT370077, KT370066, KT370087
A/Environment/Guangdong/QY208/2014(H5N6)	QY208	Qingyuan	2014.05	KT370110, KT370103, KT370096, KT370058, KT370083, KT370073, KT370068, KT370089
A/Environment/Guangdong/ZS356/2014(H5N6)	ZS356	Zhongshan	2014.07	KT370107, KT370100, KT370093, KT370061, KT370080, KT370078, KT370065, KT370086
A/Environment/Guangdong/HY243/2015(H5N6)	HY243	Heyuan	2015.02	KT370112, KT370104, KT370097, KT370063, KT370085, KT370072, KT370070, KT370090
A/Environment/Guangdong/GZ670/2015(H5N6)	GZ670	Guangzhou	2015.10	KU852961, KU852958, KU852955, KU852946, KU852952, KU852949, KU852964, KU852965
A//Environment/Guangdong/GZ693/2015(H5N6)	GZ693	Guangzhou	2015.11	KU852959, KU852956, KU852953, KU852944, KU852950, KU852947, KU852962, KU852966
A/Environment/Guangdong/ZS558/2015(H5N6)	ZS558	Zhongshan	2015.05	KU852960, KU852957, KU852954, KU852945, KU852951, KU852948, KU852963, KU852967

## Results

### Prevalence of the H5-subtype AIV in LPMs

From April 2013 to December 2015, a total of 32,452 fecal and swabs were collected from LPMs in 21 cities in Guangdong Province (Table 2). Among all of the samples, 6865 (21.2%) were positive for influenza A, 14.6% of which with the H5 subtype. The H5N1 subtype was the most prevalent among the H5 subtypes, followed by H5N6; also observed were H5N2, H5N3, H5N4, H5N5, H5N7, H5N8, and H5N9. During the same period, we selected 10 H5N6 subtypes among the 66 H5N6-positive samples in different cities of Guangdong Province in different years to analyze the evolution of the subtype (Table 1).

**Table 2:** **Environment surveillance of H5-subtype avian influenza viruses in Guangdong, 2013–2015**.

**City**	**2013**			**2014**			**2015**		
	**No. of samples collected**	**No. of positive samples collected**		**No. of samples collected**	**No. of positive samples collected**		**No. of samples collected**	**No. of positive samples collected**	
		**Total**	**H5**		**Total**	**H5**		**Total**	**H5**
Chaozhou	–^a^	–	–	5	–	–	582	96	0
Dongguan	321	37	23	588	24	13	4305	358	96
Foshan	31	2	0	464	121	12	1837	412	43
Guangzhou	274	2	2	655	39	4	169	90	0
Heyuan	–	–	–	301	13	5	801	65	30
Huizhou	–	–	–	80	56	11	350	141	12
Jiangmen	–	–	–	690	204	17	1292	376	43
Jieyang	–	–	–	311	190	49	683	215	22
Maoming	157	18	3	337	121	18	663	167	29
Meizhou	101	5	0	1050	121	10	1951	187	4
Qingyuan	–	–	–	240	84	13	604	244	27
Shantou	–	–	–	364	136	11	747	63	0
Shanwei	–	–	–	64	14	5	421	35	0
Shaoguan	–	–	–	105	35	1	535	105	7
Shenzhen	196	48	14	384	151	25	621	57	0
Yangjiang	343	69	–	1344	214	3	921	104	10
Yunfu	213	41	7	374	85	5	725	142	5
Zhanjiang	–	–	–	84	32	6	415	45	5
Zhaoqing	405	108	18	110	27	2	1470	381	83
Zhongshan	–	–	–	1021	529	155	1031	324	89
Zhuhai	–	–	–	295	163	13	599	200	7
Total	2041	330	67	8866	2359	378	21,545	4176	554
		(16.2%)^b^	(20.3%)^c^		(26.6%)	(16.0%)		(19.4%)	(13.3%)

a *Undetected*.

b *Percent positives of total collected samples in a year*.

c *Percent H5 positives of total positives samples in a year*.

### Phylogenetic analysis of surface genes

The genomes of the 10 H5N6 AIVs isolated from environmental samples were sequenced by using a next-generation sequencer (Ion PGM, Life Technologies). The complete genomes of the 10 samples were compared with nucleotide sequences of some viruses in GenBank databases.

**Figure 1:**
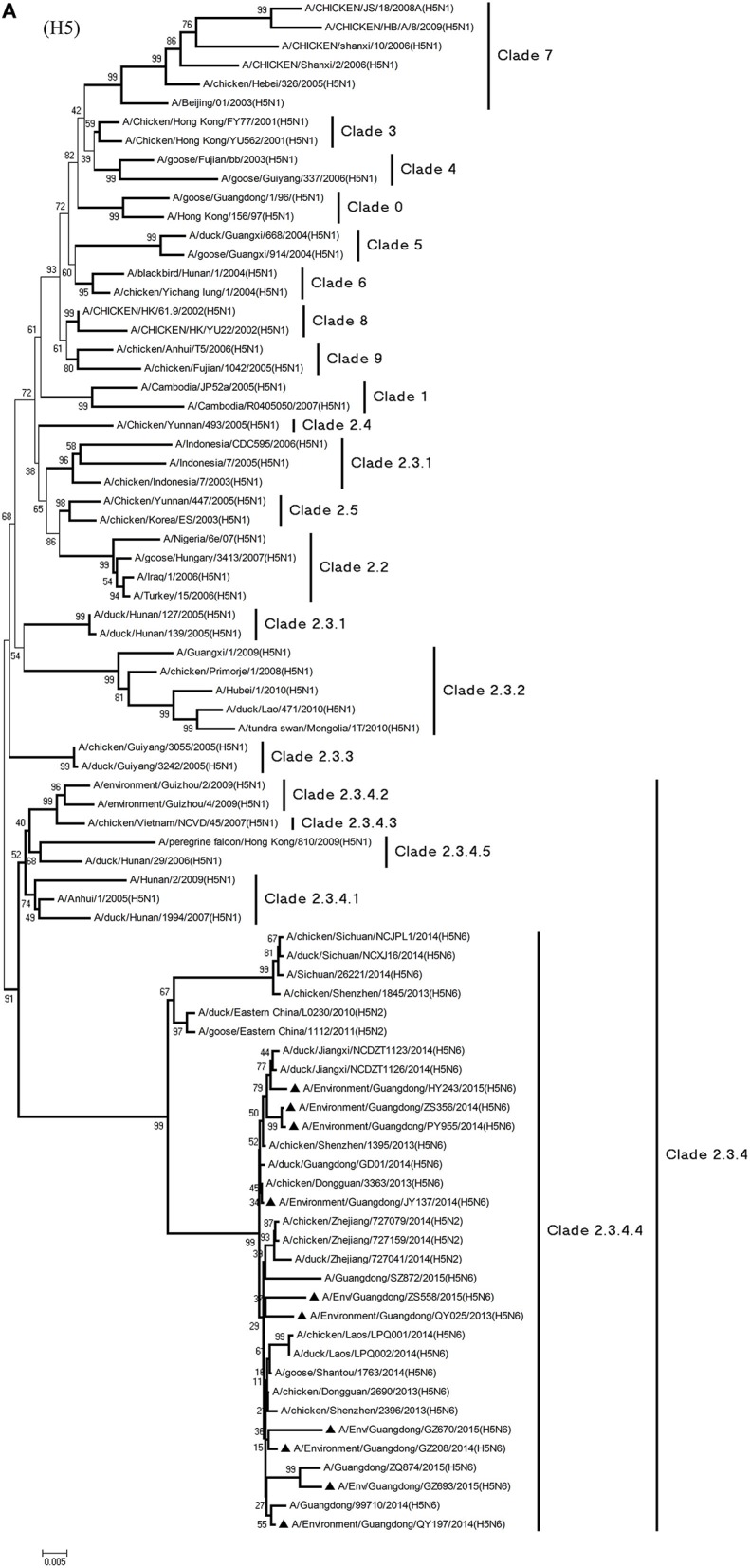

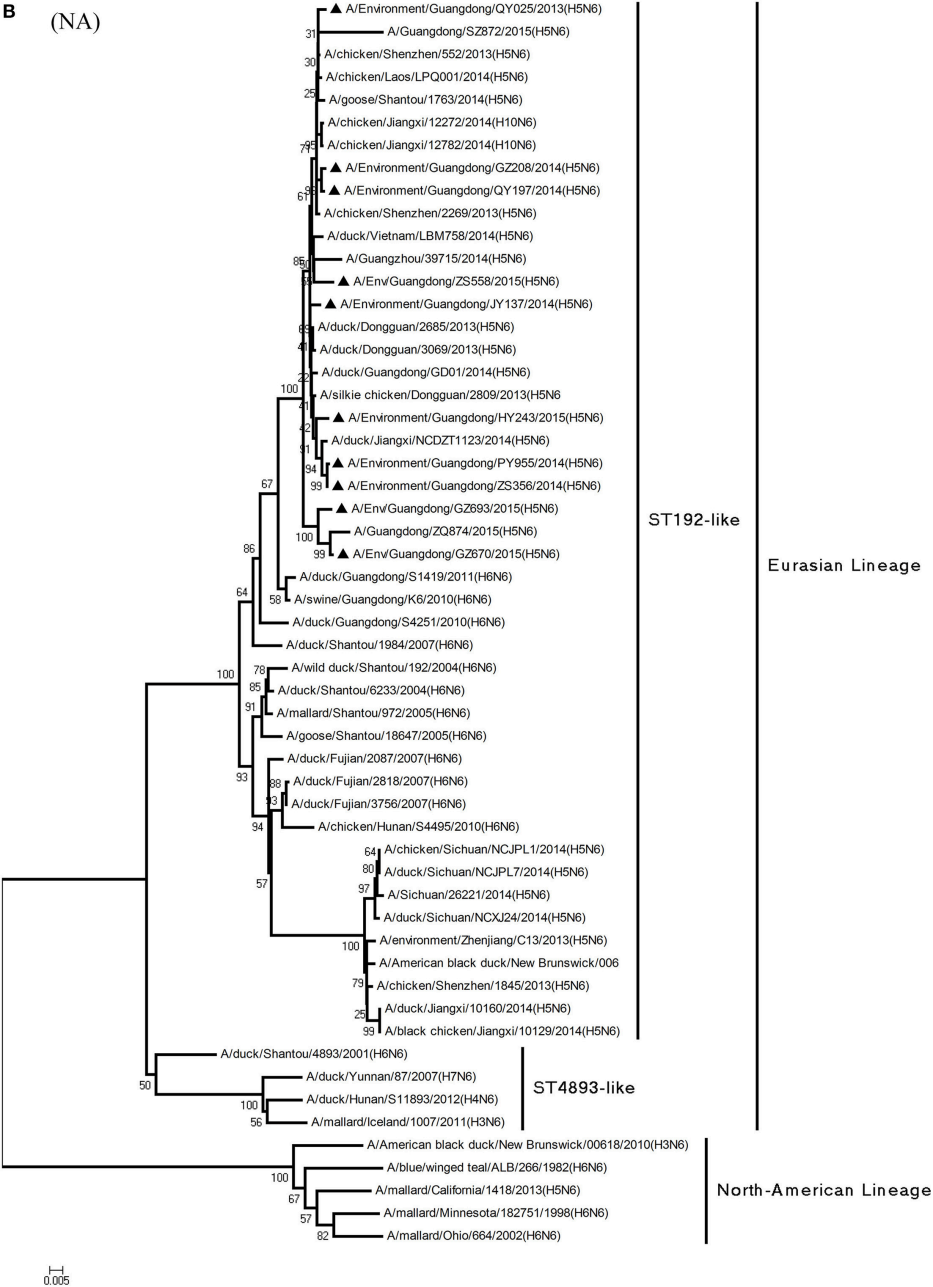

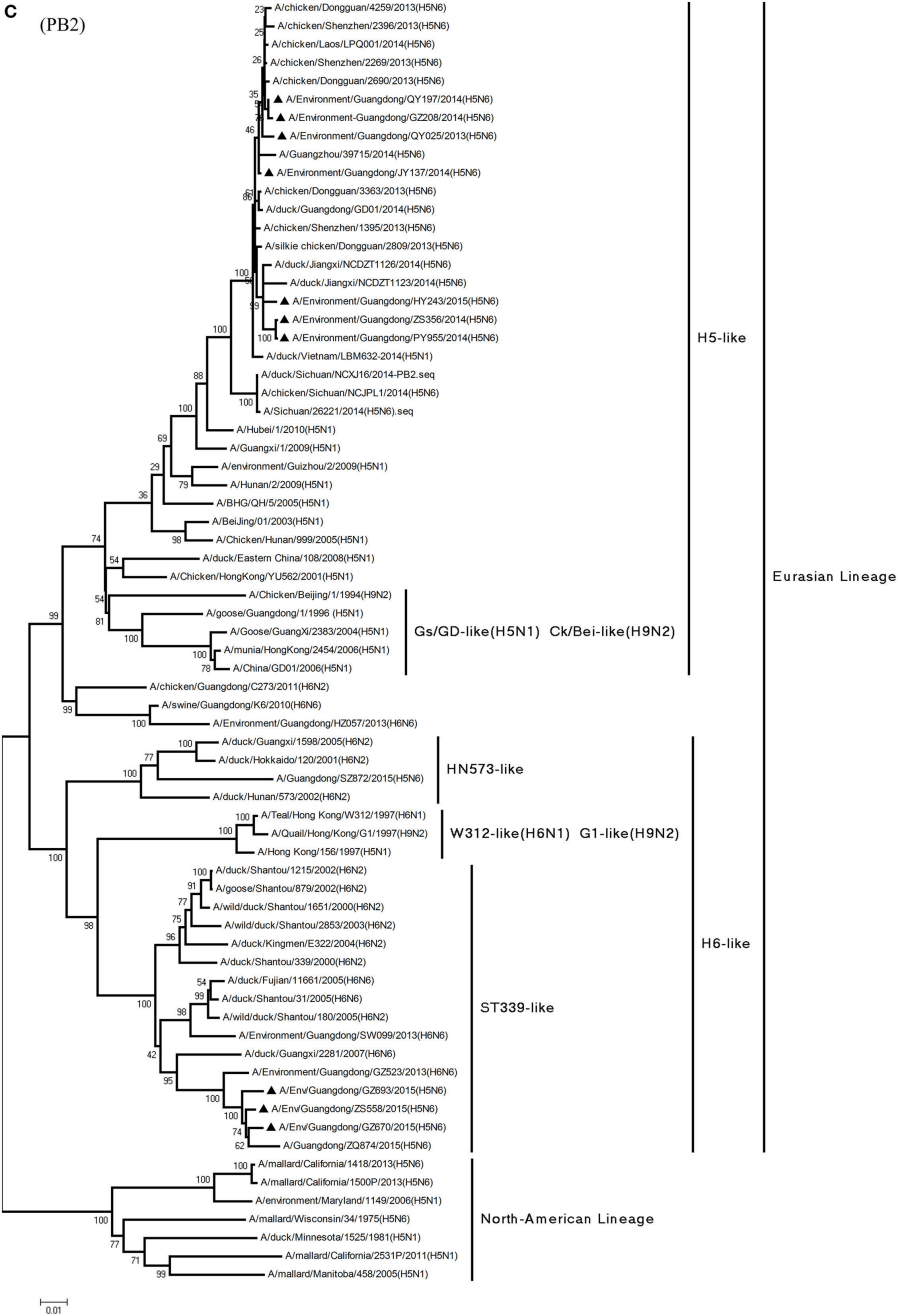

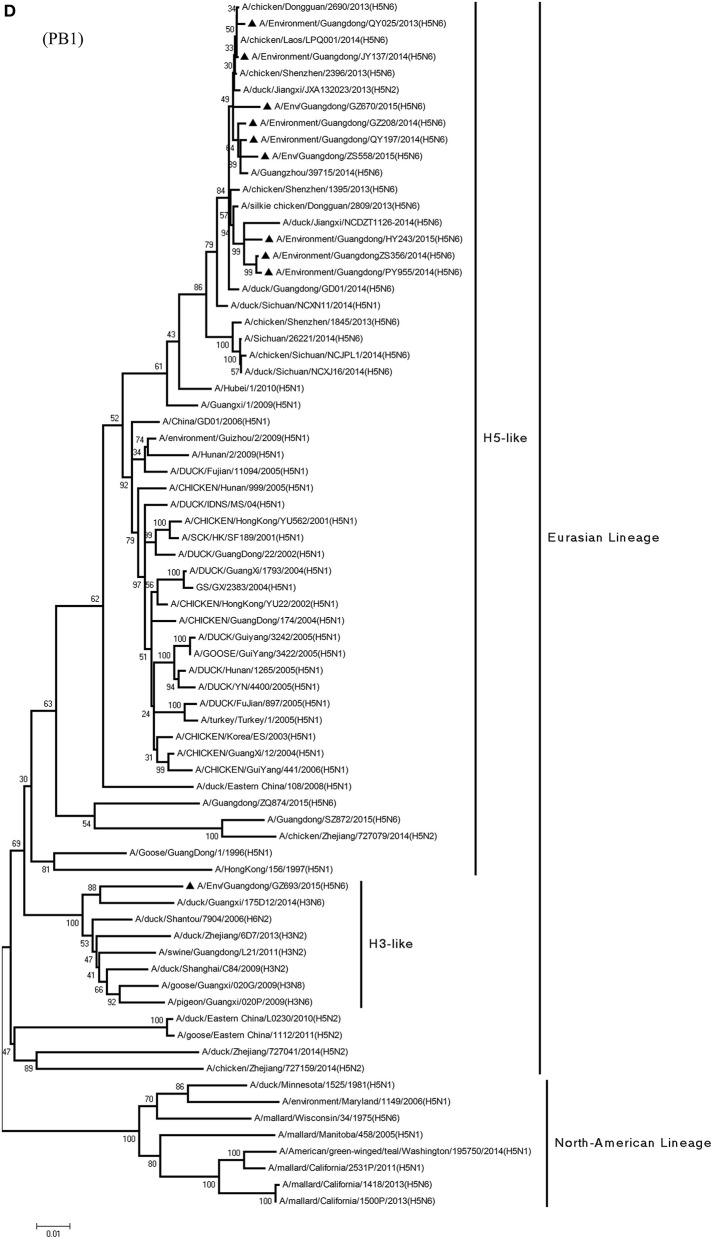

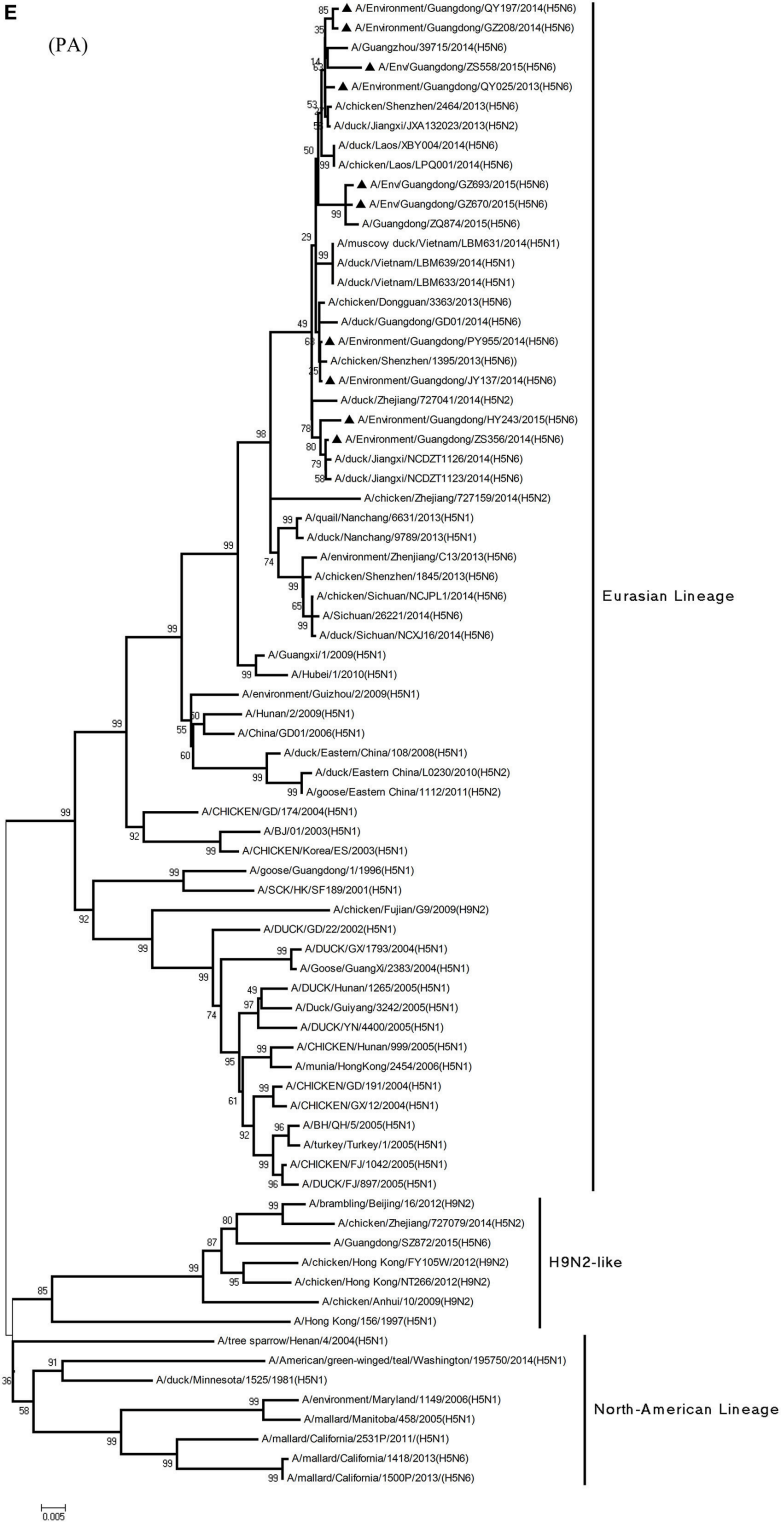

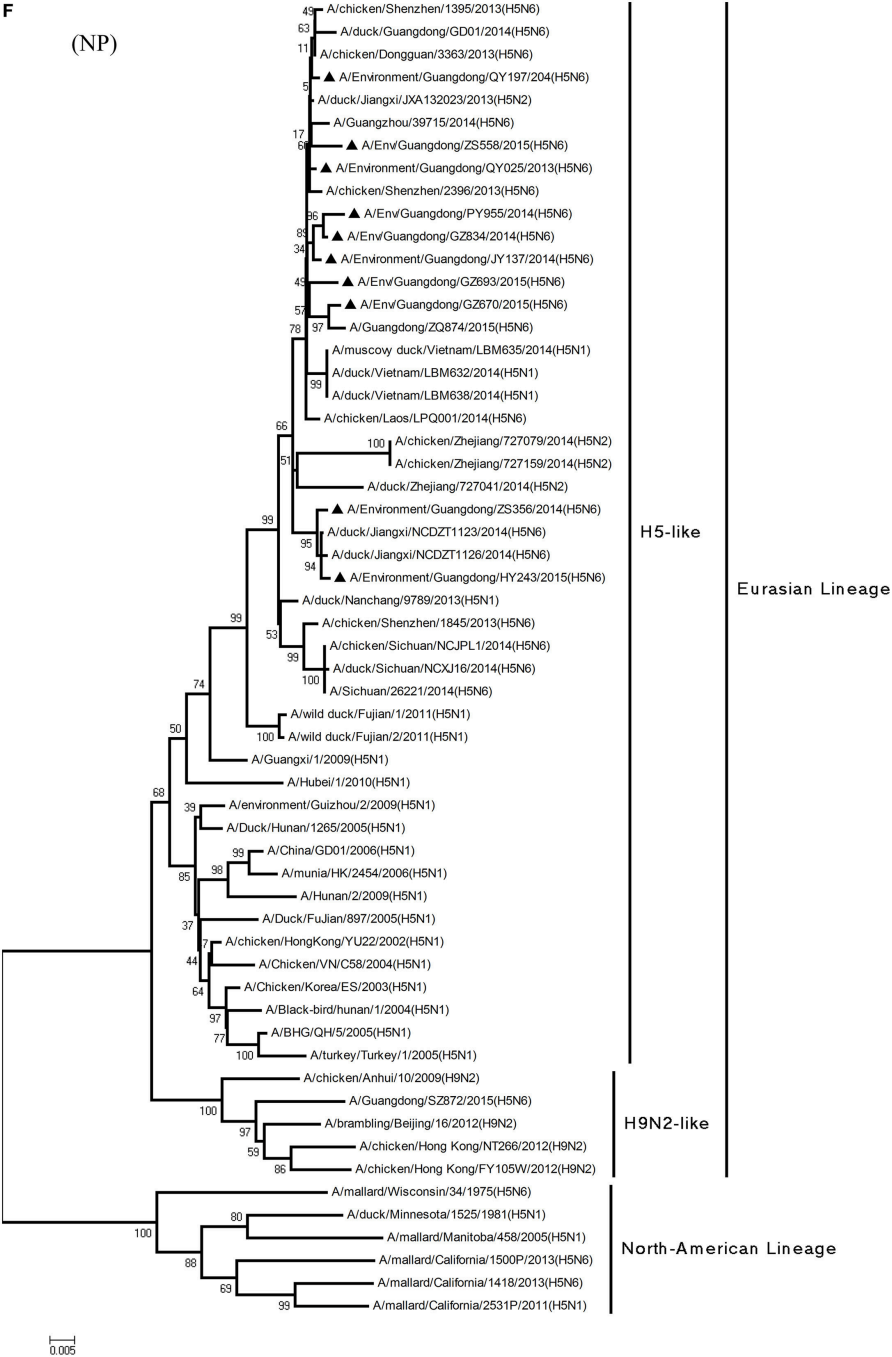

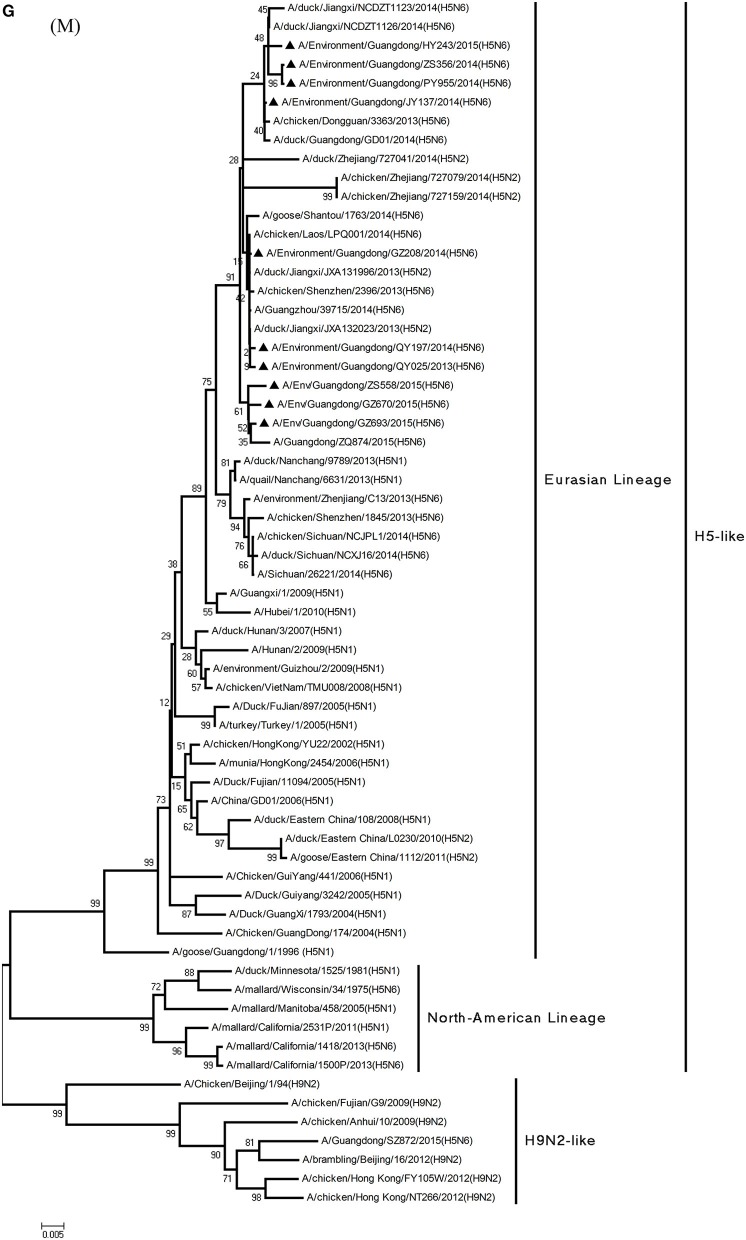

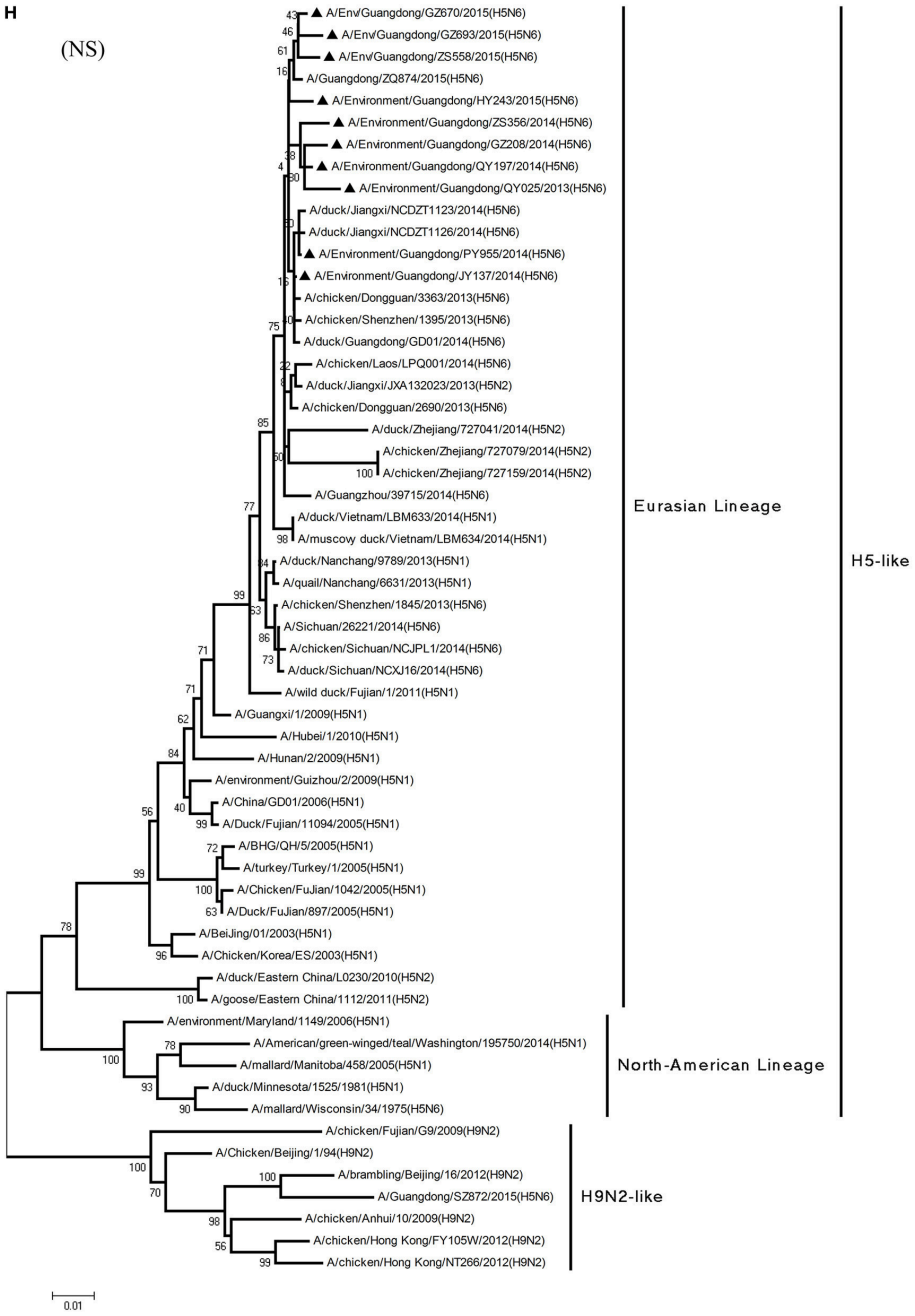
**Phylogenetic analyses of the open reading frames of H5N6-subtype avian influenza viruses**. Viruses highlighted with black triangles (▴) were characterized in the present study. The tree was constructed using the neighbor-joining of Molecular Evolutionary Genetics Analysis 6.06, with 1000 bootstrap trials to ensure confidence in the groupings.

**Figure 2:**
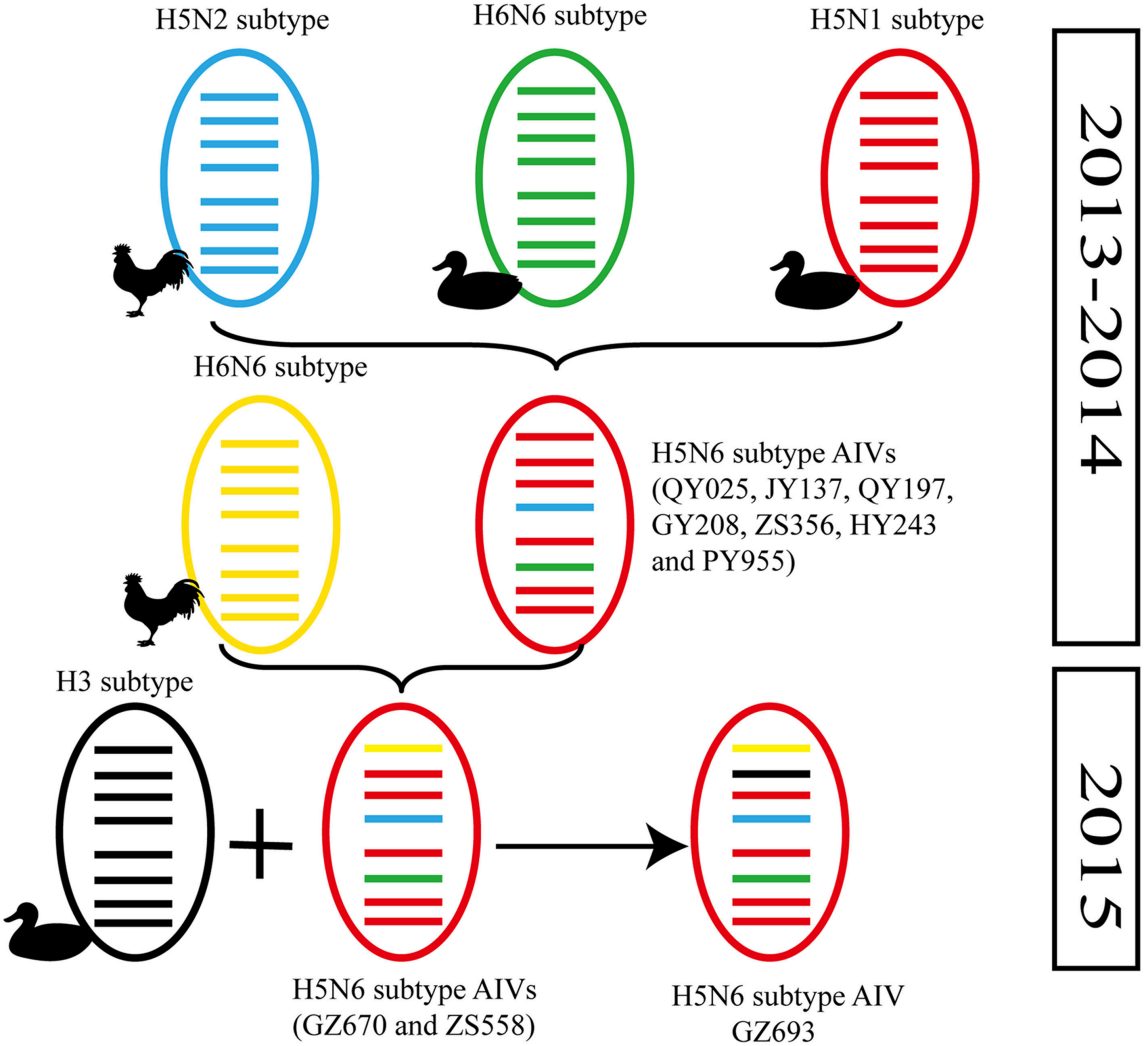
**Possible genetic contributions of known donor AIVs to the novel H5N6 avian influenza virus in Southern China**. From top to bottom, the eight genes in each schematic virus particle are the PB2, PB1, PA, HA, NP, NA, M, and NS genes. Genes of the same lineage appear in the same color.

Phylogenetic analyses demonstrated the origin and evolution of H5N6 AIVs in China. As results of the phylogenetic analysis of H5 and related viruses show, the HA gene of all 10 viruses clustered into clade 2.3.4.4 (Figure 1A) and thus related more closely to the H5N2 HPAIV, A/chicken/Zhejiang/727159/2014(H5N2), which circulates in Zhejiang Province (Figures 1A, 2). In addition, QY025, QY197, QY208, GZ670, and ZS558 shared 98.5–99.9% highest nucleotide similarity with A/chicken/Dongguan/2690/2013(H5N6) (GD-H5N6), JY137, PY955, and ZS356 shared 99.1–99.6% highest nucleotide similarity with A/chicken/Shenzhen/1395/2013(H5N6) (GD-H5N6), and HY243 shared 99.1% highest nucleotide similarity with JX-H5N6. More singularly, GZ693 shared 98.7% highest nucleotide similarity with A/Guangdong/ZQ874/2015(H5N6) (ZQ874), which was found to have recently infected human in Zhaoqing, Guangdong Province.

Phylogenetic analysis of the N6-NA gene indicated that it likely originated in H6N6 AIVs found in domestic ducks in southern China (Huang et al., 2012). According to their geographical location, the HA genes of H6-subtype AIV can be identified as either of Eurasian or North American lineage. As Figure 1B shows, those of Eurasian lineage can be divided into two groups: Group 1 (ST192-like) and Group 2 (ST4893-like) (Huang et al., 2012). All 10 viruses were of Group 1 of Eurasian lineage, represented by A/wild duck/Shantou/192/2004 (H6N6) (Figures 1B, 2). QY025, QY197, and QY208 shared 97.2–98.8% nucleotide similarity with A/chicken/Shenzhen/552/2013(H5N6) (GD-H5N6), JY137, ZS356, HY243, and ZS558 shared 96.5–99.6% highest nucleotide similarity with A/chicken/Dongguan/2685/2013(H5N6) (GD-H5N6), PY955 shared 99.6% highest nucleotide similarity with JX-H5N6, and GZ670 and GZ693 shared 96.0–96.9% highest nucleotide similarity with ZQ874 isolated from a patient in Guangdong. In all, these results suggest that the surface genes of the 10 reassortant viruses derived from H5 and H6 AIV subtypes circulating in poultry in China.

### Phylogenetic analysis of internal genes

Phylogenetic analyses of internal genes showed that the PB2, PB1, PA, M, and NS genes of all 10 viruses were of Eurasian lineage (Figures 1C–H). H5N6 AIVs did not cluster with H5N1 AIVs, but formed an independent lineage (Figure 1).

In the PB2 gene, QY025, QY197, QY208, JY137, and ZS356 shared 99.1–99.9% highest nucleotide similarity with GD-H5N6, whereas ZS356, PY955 and HY243 shared more than 99.4% highest nucleotide similarity with JX-H5N6. GZ670, GZ693, and ZS558 originated from H6-subtype AIVs and were ST339-like viruses, represented by A/wild duck/Shantou/339/2000 (H6N2) (Figure 1C).

From the PB1 gene, PY955 and HY243 shared 99.0–99.1% highest nucleotide similarity with DG-H5N6, whereas the other seven viruses shared more than 98.8% highest nucleotide similarity with Vietnam-H5N6. Meanwhile, the PB1 gene of GZ693 shared highest nucleotide similarity with H3-subtype AIVs.

Regarding the PA gene, ZS356, and HY243 shared 99.7–99.3% highest nucleotide similarity with JX-H5N6, whereas the other eight viruses shared more than 99.3% highest nucleotide similarity with GD-H5N6. As for the NP gene, PY955, ZS356, and HY243 shared more than 99.7% highest nucleotide similarity with JX-H5N6, though the other seven viruses shared 99.4–99.8% highest nucleotide similarity with GD-H5N6, and concerning the M gene, all 10 viruses shared 99.6–100% highest nucleotide similarity with JX-H5N6. Lastly, regarding the NS gene, PY955 and JY137 shared 97.6–99.9% highest nucleotide similarity with JX-H5N6, whereas the other eight viruses shared 98.7–99.4% highest nucleotide similarity with GD-H5N6.

In particular, the 10 environmental viruses shared more than 96.0% high nucleotide similarity with the H5N6 AIVs isolated from patients in Guangdong. Phylogenetic analysis demonstrated that the internal genes of seven AIVs isolated within the first 7 months of 2014 related more closely to H5N1 HPAIVs circulating in poultry in China. By contrast, GZ670, GZ693, and ZS558 isolated in 2015 diverged from previously sequenced H5N6 AIVs and related more closely to H6N2 AIVs in the PB2 gene (Figure 2).

### Molecular characterization

The HA gene of all 10 H5N6 AIVs showed the HPAIV amino acid sequence RERRRKR↓G at the cleavage site of HA1 and HA2. Amino acid residues Q226 and G228, according to H3 numbering, occurred in the receptor-binding pocket of HA1, thus indicating that the viruses preferred to bind to the AIV receptor (Ha et al., 2001). Each of the 10 AIVs had six potential N-linked glycosylation sites at HA1 (26 or 27, 39, 181, 209, and 302) and two in HA2 (499 and 558). However, ZS558 revealed A254T mutation in an extra potential glycosylation site, whereas GZ693 exhibited six potential N-linked glycosylation sites in HA1 (i.e., at positions 27, 39, 180, 208, 230, and 301) and two in HA2 (i.e., at positions 498 and 557).

The NA proteins of JY137 and PY955 exhibited 12 amino acid deletion residues (i.e., at positions 59–70) in the neck, which could boost its virulence in mammals (Matsuoka et al., 2009). The key antiviral neuraminidase inhibitor drugs sites of the NA and M genes, such as position H275 of the NA gene (NA of GS/GD number) and position S31 of the M gene, showed no mutations (Scholtissek et al., 1998; Suzuki et al., 2003).

The PB2 gene of the 10 isolated viruses was E at position 627 and D at position 701, which indicates that all isolated viruses derived from avian sources (Li et al., 2005). At the same time, all environmental viruses were M at position 317 of the PB1 protein, which implies that they are hardly either pathogenic or non-pathogenic to mice (Katz et al., 2000). The AIVs could suppress a host's antiviral defenses relative to the antiviral effects of cytokines such as interferon. All viruses had P42S and D92E mutations in the NS1 protein, which suggests that they could enhance resistance to cytokines (Jiao et al., 2008; Qi et al., 2009).

## Discussion

At present, H5N1 AIVs have become endemic in waterfowl and domestic poultry in China, Southeast Asia, North America, and Africa, where they have evolved into multiple phylogenetic lineages (WHO/OIE/FAO, 2012). The regular transmission of H5N1 HPAIVs among waterfowl and domestic poultry has facilitated genetic diversity among circulating clades in poultry in China (Duan et al., 2008; Vijaykrishna et al., 2008). In particular, the AIVs of clades 2.3.2, 2.3.4, and 7.2 have cocirculated predominantly in domestic poultry and waterfowl in China continuously since 2007 (Smith et al., 2009; Jiang et al., 2010; Li et al., 2010). At the same time, evolutionary clades such as 2.3.4.5 and 2.3.4.6—recently redefined as clade 2.3.4.4—have been reported (Gu et al., 2013). Moreover, H5-subtype AIVs from clade 2.3.4.4 appear to be gradually replacing AIVs from clade 2.3.4.2, especially in waterfowl. In March 2014, an emergent H5N6 AIV caused an outbreak in poultry in Laos (Wong et al., 2015), and later, a flock of ducks was infected with H5N6 AIVs in Guangdong Province (Shen et al., 2015). Genetic analysis suggested that subtype H5N6 AIVs originated from clade 2.3.2.1b and variant clade 2.3.4 in H5N1 AIVs (Shen et al., 2015; Wong et al., 2015). As phylogenic analysis shows, of the 10 H5N6 AIVs isolated as part of LPM surveillance during 2013–2015, all environmental samples belonged to novel clade 2.3.4.4 and probably evolved to form a new subcluster, unlike those of H5N6 s previously identified in Sichuan Province.

Alongside HA evolution, the NA gene of H5N1 AIV has frequently reassorted with other subtypes of AIVs circulating in poultry (Zhao et al., 2008; Neumann et al., 2010). The new reassortments, including H5N3, H5N6, and H5N8, together with H7N9 and H9N2, are currently cocirculating in domestic poultry and waterfowl worldwide. In our study, H5N6 AIVs were natural recombinants, the NA gene of which derived from H6N6 AIVs circulating broadly in ducks in southern China. Within the first 7 months of 2014, internal genes of H5N6 reassortants were derived from the genetic backbone of the H5N1 subtype (Wu et al., 2015). Interestingly, for H5N6 viruses isolated after 2015, we noted the divergence of three H5N6 reassortants—namely, GZ670, GZ693, and ZS558—isolated after 2015 (Figure 2). The PB2 genes of GZ670, GZ693, and ZS558 were not grouped into the same clusters as other reported H5N6 viruses, but within the same clusters as H6N2 AIVs. Furthermore, the PB1 gene of GZ693 was clustered as a H3-subtype AIV. These results indicate that H5N6 AIV is constantly evolving, and as such, novel AIVs possessing H5- and H6-derived internal genes and other AIVs possessing specific mammal-derived mutations could enhance virulence and transmissibility in humans.

After December 2014, the first H5N6 AIV infections in humans in Guangdong Province seemed to an appeared to stop. From December 2015 to January 2016, however, five H5N6 AIV infections in humans were reported in Guangdong Province (WHO, 2014b, 2016a,b,c). Consistent with the evolution of H5N6 AIVs isolated from LPMs, the sequences of H5N6 AIVs isolated from patients are constantly evolving. The whole gene sequences of the first human H5N6 AIV were similar to those of the H5N6 AIVs isolated in early 2015 in LPMs in Guangdong Province. Meanwhile, the whole gene sequences of the other four human H5N6 AIVs were consistent with those of H5N6 AIVs isolated from LPMs in late 2015. Molecular characterization and phylogenetic analysis exhibited a highly close genetic relationship between the viruses isolated from humans and LPMs, thereby suggesting that infection in humans might be caused by the LPM environment.

LPMs have been deemed potential hotbeds for infection with H5N1 and H7N9 AIVs in humans (Wan et al., 2011; Shi et al., 2013). Some human–human transmission of AIVs (e.g., H5N1 and H7N9) has been reported (Wang et al., 2008; Qi et al., 2013), and as of February 2016, nine confirmed human infections with subtype H5N6 had occurred in China's Sichuan, Guangdong, and Yunnan Provinces (CDC China, 2014; WHO, 2015b,c, 2016a,b,c). In particular, the patient infected with H5N6 AIV in Guangzhou had visited an LPM before the onset of illness and could have acquired the infection there (Yang et al., 2015). The other patient infected with H5N6 AIV and who died in Sichuan Province was a merchant at a local LPM. Moreover, the other seven cases of infection had visited LPMs in the past. Perhaps above all, we isolated 10 H5N6 AIVs in LPMs, which indicates that LPMs are potential sources of AIV infection in humans.

In conclusion, we analyzed the evolution of H5N6 samples isolated from LPM environments. Epidemiological and experimental data suggest that the H5N6 subtype currently has a limited capacity for chicken–human or environment–human transmission. LPMs can provide sufficient opportunities for close contact among waterfowl, domestic poultry, mammals, and humans, as well as potential AIV infection, which in turn results in the emergence of novel AIVs. Large-scale surveillance of LPMs therefore continues to be essential to identifying novel reassortants and sequence mutations among existing AIV subtypes.

## Author contributions

Conceived and designed the experiments: RY, CK. Performed the experiments: RY, ZW, JW, LL. Analyzed the data: RY. Contributed reagents/materials/analysis tools: RY, LZ, YS, HN, JL, XZ, CK. Wrote the paper: RY, YK.

### Conflict of interest statement

The authors declare that the research was conducted in the absence of any commercial or financial relationships that could be construed as a potential conflict of interest.
